# Integrative Assessment of *Glycyrrhiza uralensis* Extract in Cosmetics Using HPLC Analysis, Network Pharmacology, and Computational Threshold of Toxicological Concern-Based Safety Evaluation

**DOI:** 10.3390/ijms262311677

**Published:** 2025-12-02

**Authors:** Hiyoung Kim, Kihoon Park, Young Bong Kim, Minjee Kim

**Affiliations:** 1 School of Advanced Biotechnology, Konkuk University, Seoul 05029, Republic of Korea; reihyoung@konkuk.ac.kr (H.K.); kimera@konkuk.ac.kr (Y.B.K.); 2 KR Biotech, KU Technology Innovation Hall, Konkuk University, Seoul 05029, Republic of Korea; whitesoul623@krbiotech.com

**Keywords:** network pharmacology, HPLC, threshold of toxicological concern, *Glycyrrhiza uralensis*, in silico toxicology

## Abstract

Licorice *(Glycyrrhiza uralensis)* contains bioactive flavonoids and saponins, primarily liquiritin and glycyrrhizin, which exhibit pharmacological activities but also potential dose-dependent toxicity. This study aimed to establish an integrative workflow combining analytical chemistry, network pharmacology, and computational toxicology to evaluate the skin-related safety of these compounds. High-performance liquid chromatography (HPLC) was employed to quantify liquiritin and glycyrrhizin in licorice extract. Network pharmacology and molecular docking analyses were conducted to identify core toxicity-related targets. In silico toxicity and threshold of toxicological concern (TTC) assessments were performed using VEGA and database-driven prediction models to estimate dermal exposure risk. Liquiritin and glycyrrhizin were identified as major constituents of *G. uralensis*. Network analysis revealed three key targets—EGFR, STAT3, and SRC—linked to skin sensitivity and toxicological pathways, including TRP channel regulation and EGFR signaling. Molecular docking showed strong binding affinities to SRC. The threshold of toxicological concern evaluation indicated that liquiritin exposure remained below safety thresholds, while glycyrrhizin slightly exceeded but remained within acceptable limits. The proposed HPLC–network pharmacology–TTC workflow provides a novel, non-animal approach for early-stage cosmetic safety screening. Both compounds demonstrate acceptable safety margins, supporting their controlled use in dermal formulations.

## 1. Introduction

Licorice (*Glycyrrhiza uralensis,* GU) extracts are widely incorporated into both cosmetic formulations and functional foods due to their pharmacological versatility, including anti-inflammatory, antioxidant, and skin-whitening properties [1,2]. However, despite the long-standing traditional use of licorice, systematic toxicological data supporting its safety under modern conditions of use remain incomplete [3]. From a cosmetic perspective, chronic dermal application of licorice extracts raises safety concerns, including the potential for endocrine disruption, skin sensitization, and prooxidant effects, particularly when bioactive flavonoids are used in skin-lightening or anti-aging formulations [4]. GU contains a broad spectrum of bioactive constituents, among which liquiritin and glycyrrhizin are regarded as principal molecular markers. Their levels vary substantially with plant origin and extraction conditions, with glycyrrhizin typically reported at 1–8% and liquiritin at 0.1–4% of the extract [5,6,7,8]. Both compounds have been extensively studied for their pharmacological effects, including anti-inflammatory, hepatoprotective, and cardioprotective properties [9,10,11]. However, their therapeutic potential does not negate toxicological concern. Glycyrrhizin, in particular, is associated with well-characterized adverse effects—including hypertension, hypokalemia, and edema—mediated through pseudoaldosteronism-like interference with mineralocorticoid pathways [12]. Although provisional safety thresholds exist, they do not fully account for variability linked to metabolic differences or formulation-dependent bioavailability. Recent metabolomic studies have further clarified the molecular perturbations underlying glycyrrhizin-induced toxicity, providing refined mechanistic insight and potential predictive biomarkers [13,14]. Liquiritin is also widely used as a functional licorice flavonoid, yet its toxicological evidence—particularly regarding dermal exposure—remains limited, underscoring the need for more systematic safety evaluation [15].

In recent years, the integration of network toxicology and molecular docking has emerged as an effective strategy for elucidating mechanisms of toxicity and predicting compound–target interactions [16]. The concept of network toxicology represents an important extension of network pharmacology into the field of toxicological research. Network toxicology combines principles of network pharmacology and systems biology, providing a holistic framework to investigate toxic effects at the molecular, cellular, and systemic levels. By incorporating bioinformatics, big data analytics, genomics, proteomics, and metabolomics, this approach enables comprehensive characterization of toxicity mechanisms and facilitates the identification of key molecular pathways involved in adverse biological responses. In silico toxicology is increasingly recognized as an efficient tool for modern risk assessment, allowing the prediction of toxicity without extensive animal testing [17]. It accelerates safety evaluations, supports the 3Rs principle (Replacement, Reduction, and Refinement), and integrates toxicological databases to provide a cost-effective and ethical framework for hazard identification in pharmaceuticals, cosmetics, and food applications [18]. Computational methods, including predictive platforms, enable early hazard identification and prioritization of compounds for further testing [19]. These methods are particularly suited for complex phytochemical mixtures, where experimental data may be scarce or inconsistent.

The threshold of toxicological concern (TTC) concept, combined with the Cramer classification scheme, offers a pragmatic framework for the safety evaluation of licorice-derived compounds [20,21]. By assigning each constituent to structural classes associated with conservative threshold of toxicological concern values, it is possible to establish acceptable exposure thresholds for cosmetic and food applications [19,21]. Integration with validated animal alternative methods, such as OECD-endorsed in vitro and in chemico assays, further strengthens the regulatory acceptance of such approaches [22].

In this context, the objective of this present study was to (i) retrieve main compounds of GU from HPLC analysis, (ii) predict toxic targets from network pharmacology, and (iii) conduct risk and safety assessment using in silico toxicology tools and the threshold of toxicological concern concept in cosmetic application scenarios. While preliminary, the presented framework integrates multi-tiered toxicity endpoints into a unified screening pipeline. This integrative approach is aligned with the *OECD* and *EFSA* recommendations for new-approach methodologies (NAMs) in predictive toxicology, which emphasize the use of computational evidence for early-stage chemical risk assessment and reduction of unnecessary animal testing. We aim to identify potential risks of natural products at an early stage, while aligning with global trends in cosmetic safety assessment that emphasize non-animal testing, and evidence-based risk management.

## 2. Results

### 2.1. Quantitative Analysis of Liquiritin and Glycyrrhizin

**Figure 1:**
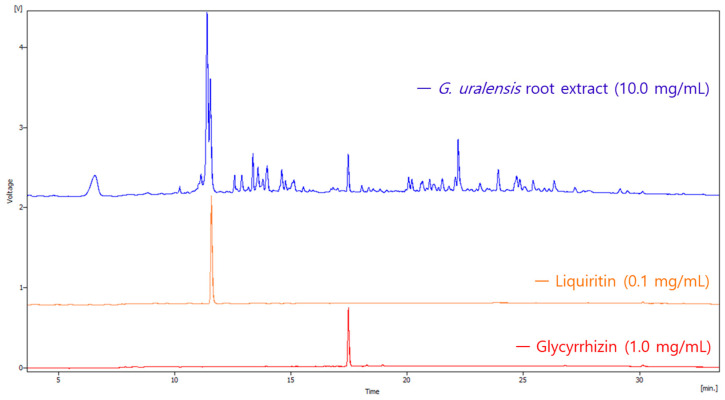
HPLC chromatograms comparing *G. uralensis* root extract (10.0 mg/mL, blue), liquiritin standard (0.1 mg/mL, orange), and glycyrrhizin standard (1.0 mg/mL, red). The retention times of liquiritin and glycyrrhizin in the extract are confirmed by alignment with the respective standards.

The HPLC chromatogram of the *Glycyrrhiza uralensis* root extract detected at 215 nm indicated the presence of multiple chemical constituents (Figure 1). Quantitative analysis was performed using authentic standards of liquiritin and glycyrrhizin. Based on HPLC analysis, the contents of liquiritin and glycyrrhizin in the extract were quantified as 1.1% and 1.2%, respectively.

### 2.2. Quantitative Validation of the Analytical Method

The linearity of detector response was evaluated for quantitative validation of compounds (Table 1). Both liquiritin and glycyrrhizin showed excellent linearity within their respective concentration ranges (liquiritin: 6.25–1250 μg/mL; glycyrrhizin: 12.5–2500 μg/mL), with correlation coefficients ($r^{2}$) of 0.9994 and 0.9997, respectively. The regression equations for calibration curves were y=573.21x−1.3025 for liquiritin and y=388.75x+0.5123 for glycyrrhizin. Sensitivity assessment yielded limits of detection (LOD) of 1.5 μg/mL for liquiritin and 3.1 μg/mL for glycyrrhizin, with limits of quantification (LOQ) of 6.25 and 12.5 μg/mL, respectively. These results confirm that the developed method is highly sensitive and reliable for the quantitative analysis of both analytes.

### 2.3. Physicochemical Descriptors of Liquiritin and Glycyrrhizin

Liquiritin and glycyrrhizin were analyzed through the SwissADME database (http://www.swissadme.ch/index.php, accessed on 1 August 2025) for Lipinski’s rule of five (RO5) (Table 2). The Lipinski guideline characterizes molecules based on physicochemical property, which include a molecular weight (MW) ≤ 500, hydrogen bond acceptor (HBA) ≤ 10, hydrogen bond doner (HBD) ≤ 5, and lipophilicity (cLogP) ≤ 5 [23,24]. Between two compounds, only liquiritin met Lipinski’s RO5. Glycyrrhizin violated MW, HBA, and HBD, suggesting a low permeability or poor absorption.

### 2.4. Identification of Targets of Liquiritin- and Glycyrrhizin-Induced Skin Sensitivity and Toxicology

**Figure 2:**
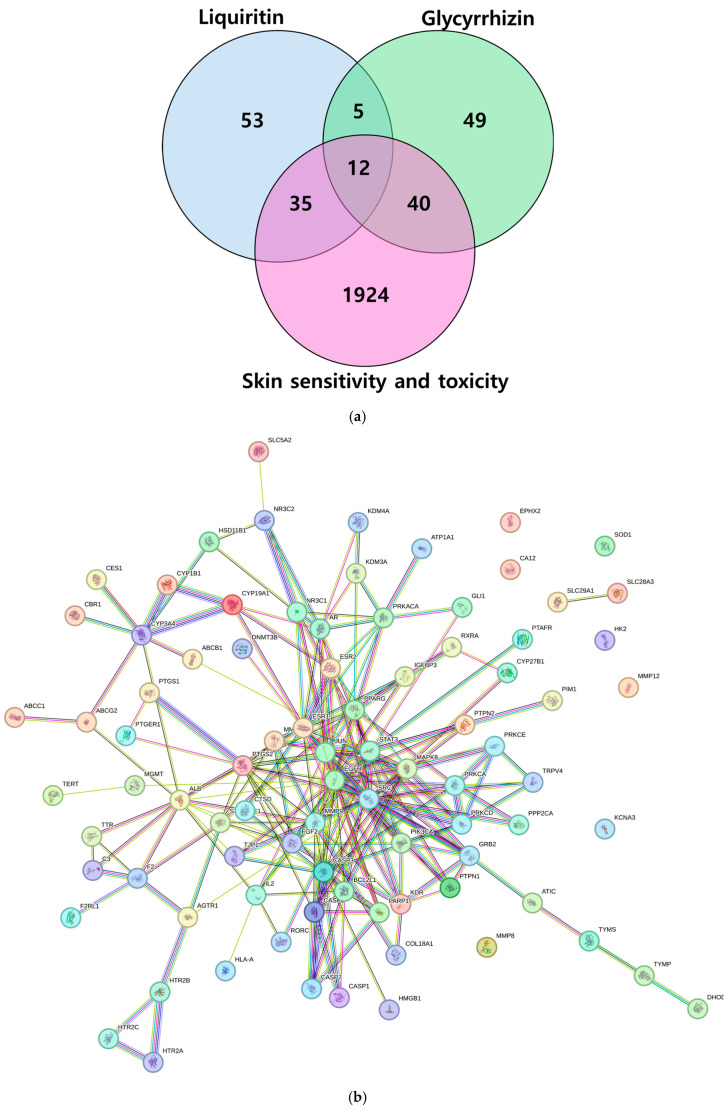
Target identification and PPI network construction. (**a**) Venn diagram of the targets of liquiritin, glycyrrhizin, and skin sensitivity and toxicity; (**b**) PPI network of hub targets constructed in STRING database and analyzed using Cytoscape ver.3.10.4.

In this study, a total of 105 targets associated with liquiritin and 106 targets related to glycyrrhizin were initially screened from the SwissTargetPrediction and DrugBank databases. From the NCBI Gene and GeneCards databases, 2011 targets linked to “skin sensitivity” and “skin toxicity” were retrieved. By intersecting these datasets, 87 core targets (35 + 12 + 40) related to skin sensitivity and toxicity were identified as potential hub targets (Figure 2). Subsequently, a protein–protein interaction (PPI) network was constructed using the STRING database, resulting in 86 nodes and 231 edges. Topological analysis revealed that the three most highly connected hub proteins were epidermal growth factor receptor (EGFR; degree = 29), signal transducer and activator of transcription 3 (STAT3; degree = 25), and proto-oncogene tyrosine-protein kinase SRC (SRC; degree = 23), indicating their central regulatory roles within the PPI network.

### 2.5. Target Function and Pathway Enrichment of Core Pathway

**Figure 3:**
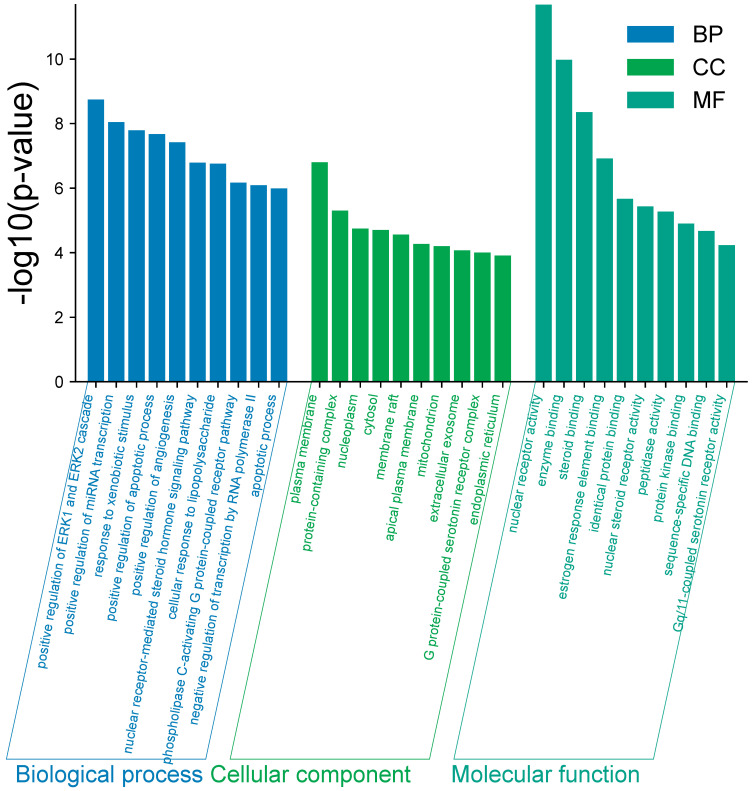
GO enrichment analysis of potential targets. The bar chart illustrates the top 10 significantly enriched Gene Ontology (GO) terms in each category—biological process (BP), cellular component (CC), and molecular function (MF)—among the 87 identified potential targets, ranked by ascending *p*-values.

**Figure 4:**
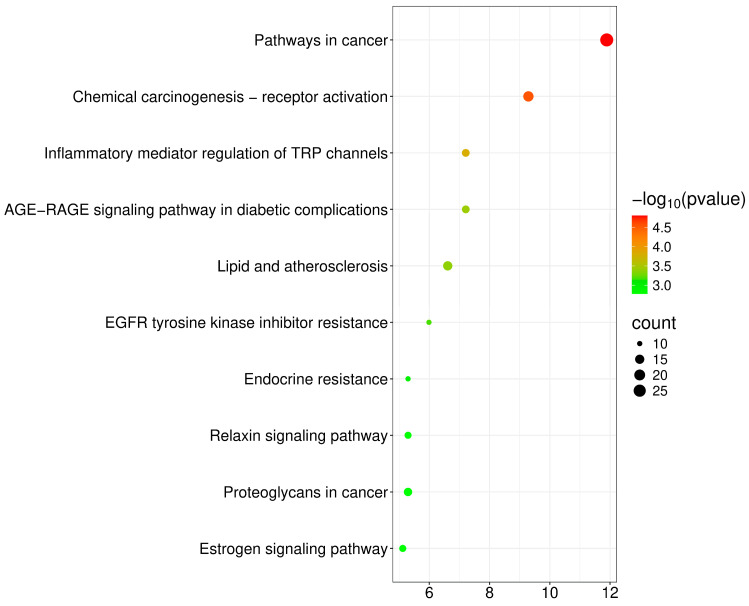
TOP 10 KEGG enrichment analysis of potential targets based on −log10 (*p*-value) and gene count.

We performed a Gene Ontology (GO) analysis of 87 potential targets using the DAVID database, restricting the species to *Homo sapiens*. The analysis yielded a total of 455 statistically significant GO entries for 308 biological processes (BP), 43 cellular components (CC), and 104 molecular functions (MF). The GO entries were ranked according to false discovery rate (FDR) values, and the top 10 entries with the lowest FDR values in BP, CC, and MF were selected and visually depicted in the enrichment analysis plot (Figure 3). KEGG analysis was performed on these potential targets using the DAVID database to identify their involvement in specific signaling pathways. Out of a total of 126 signaling pathways, we generated significance statistical bubble plots and categorical histograms (Figure 4) to visually represent the top 10 KEGG signaling pathways in terms of the reverse order log *p*-value. Based on the GO enrichment analysis of the potential targets, the identified genes were broadly distributed and predominantly expressed in the plasma membrane. Many of these targets were involved in key regulatory signaling processes, including the ERK1/ERK2 cascade and apoptotic pathways, suggesting their participation in cell survival and stress response mechanisms. KEGG pathway enrichment analysis revealed significant associations with signaling pathways related to skin sensitivity and toxicity. Notably, enriched pathways included inflammatory mediator regulation of TRP channels, EGFR tyrosine kinase inhibitor resistance, the relaxin signaling pathway, and several cancer-related signaling routes. These findings collectively align with the observed skin sensitivity and toxicological effects induced by liquiritin and glycyrrhizin, highlighting their potential mechanistic relevance in epidermal and inflammatory responses.

### 2.6. Prediction of Toxic Targets of Liquiritin and Glycyrrhizin and Molecular Docking Analysis

The molecular docking analysis was performed to evaluate the interactions between liquiritin, glycyrrhizin, and the three core target proteins identified in the network analysis: epidermal growth factor receptor (EGFR; degree = 29), signal transducer and activator of transcription 3 (STAT3; degree = 25), and proto-oncogene tyrosine-protein kinase SRC (SRC; degree = 23) (Table 3). Both liquiritin and glycyrrhizin exhibited favorable binding affinities with these core targets. Among them, SRC demonstrated the strongest interaction, with binding energies of −9.8 kcal/mol for liquiritin and −9.6 kcal/mol for glycyrrhizin, indicating a high degree of binding stability and potential regulatory significance in mediating skin-related toxicological effects.

**Figure 5:**
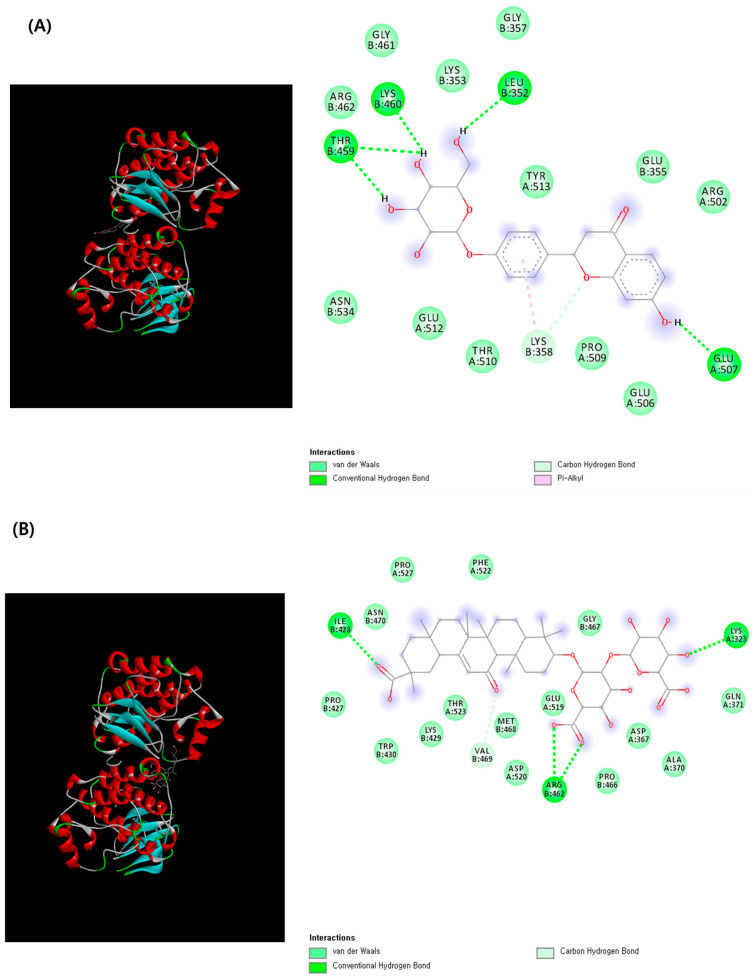
3D- and 2D-molecular docking analysis of (**A**) liquiritin–SRC and (**B**) glycyrrhizin–SRC complexes.

Ligand–receptor interactions were visualized using Discovery Studio Visualizer (Figure 5). The 2D interaction map of liquiritin–SRC revealed that the complex was stabilized through the formation of one carbon–hydrogen bond and a π–alkyl interaction with LYS358, along with four conventional hydrogen bonds involving LEU352, LYS460, THR459, and GLU507. Similarly, the glycyrrhizin–SRC complex exhibited strong stabilization through four hydrogen bonds with key amino acid residues, including ILE428, two bonds with ARG462, and one with LYS323. These interactions indicate that both compounds form energetically stable complexes with SRC, suggesting potential inhibitory activity and structural compatibility with the protein’s binding pocket.

### 2.7. In Silico Skin Sensitivity and Irritation Prediction

The in silico prediction of skin sensitivity and irritation potential was performed using the VEGA platform (version 1.2.4), employing the CONCERT model to assess dermatotoxicity endpoints (Table 4). This model integrates quantitative structure–activity relationship (QSAR) algorithms and molecular descriptor-based predictions to evaluate the likelihood of a compound eliciting skin sensitization or irritation responses.

Both liquiritin and glycyrrhizin were predicted to be “inactive” in skin sensitization, indicating a low probability of inducing allergenic reactions upon dermal exposure. In addition, both compounds were classified as “non-irritating,” suggesting minimal potential to trigger inflammatory or irritant responses on the skin surface. These findings imply a favorable dermatological safety profile for liquiritin and glycyrrhizin, consistent with their traditional use in topical and cosmetic formulations.

### 2.8. Cramer Classification and Safety Assessment Using the Threshold of Toxicological Concern Approach

For Cramer class assignment for two constituents, we utilized Toxtree 3.1 package [25]. Both liquiritin and glycyrrhizin were assigned as class III from the database. We applied the lowest threshold of toxicological concern value, i.e., 1.5 μg/kg bw/day for Cramer class III according to European Food Safety Authority (EFSA).

(1) $$SED=\frac{A(g/day)\times Cprod\left(\%\right)\times 10,000(\mu g/g)\times DA}{BW(kg)}$$

(2) $$C\max(\%)=\frac{\mathrm{TTC}\times \mathrm{BW}}{A\times DA\times 10,000}$$

A = daily product usage (g/day), DA = dermal absorption factor set at 0.5, Cprod = product concentration (% *w*/*w*), 10,000 μg/g converts % to µg/g (1% = 10,000 µg/g), BW = body weight (kg).

The margin of exposure was evaluated for liquiritin (Cprod = 0.011%) and glycyrrhizin (Cprod = 0.015%) in the representative cosmetic (face leave-on cream) category. The systemic exposure dose (SED) was calculated assuming 50% dermal absorption (DA), a 60 kg body weight, and daily product usage values. The threshold of toxicological concern for Cramer class III compounds (1.5 µg/kg bw/day; 90 µg/day for a 60 kg individual) was applied as the reference for risk characterization (Table 5).

For the face leave-on cream (application amount, A = 1.54 g/day), the estimated systemic exposure doses (SEDs) were 1.41 µg/kg bw/day for liquiritin and 1.93 µg/kg bw/day for glycyrrhizin. Liquiritin exposure remained below the threshold of toxicological concern, with a back-calculated Cmax of 0.0117%, indicating a low systemic exposure and favorable safety margin. In contrast, glycyrrhizin exceeded the threshold of toxicological concern threshold (TTC) by 1.29-fold.

Overall, these results indicate that liquiritin exposure from face cream formulations falls well within the threshold of toxicological concern safety limit, supporting its safe cosmetic use. Although glycyrrhizin exposure slightly surpassed the TTC threshold, the estimated concentration remains within a range considered to pose minimal toxicological risk under typical cosmetic use conditions.

## 3. Discussion

The early identification of potential risks in natural products is increasingly recognized as a critical component of modern safety assessment, particularly in the cosmetic sector where non-animal testing strategies are now widely prioritized. Given the chemical complexity of botanical extracts, their evaluation requires a framework that acknowledges the diverse interactions among multiple constituents that cannot be fully captured through isolated compounds alone. Current regulatory and scientific developments emphasize integrated workflows that combine chemical profiling, computational toxicology, and exposure-based concepts [26,27]. New-approach methodologies—including in silico prediction tools, high-content profiling, read-across frameworks, and exposure-driven concepts such as the threshold of toxicological concern—are now routinely applied to evaluate active substances within complex formulations. These models emphasize mechanistic understanding, dose–response relevance, and biologically informed risk interpretation, and have been successfully implemented for a range of botanical constituents [28]. A recent example is the safety evaluation of *Paeonia lactiflora* root extract using the threshold of toxicological concern-based framework, which demonstrates the growing applicability of exposure-driven, non-animal approaches to botanical cosmetic ingredients [29].

In this context, our approach highlights the value of incorporating HPLC profiling, in silico prediction, threshold of toxicological concern estimation, and network-level mechanistic interpretation as foundational components for evaluating multi-component plant-derived materials. Continued advancement of such multi-layered methodologies will be essential for establishing robust and scientifically coherent safety assessments for complex botanical matrices.

In silico toxicology now underpins rapid, animal-free safety screening across sectors, maturing its role in regulatory risk assessment. The database-driven prediction tools are well suited to complex botanical matrices by enabling early hazard identification, read-across, and prioritization for testing [17,30]. This study aimed to evaluate the main compounds of *Glycyrrhiza uralensis* (GU), determine their content, assess possible toxicity through a combination of in silico tools, and conduct safety assessment using threshold of toxicological concern concept. In this study, HPLC profiling revealed liquiritin and glycyrrhizin as two main constituents of the GU extract. To evaluate their toxicological relevance, physicochemical and predictive toxicology analyses were performed. Based on Lipinski’s rule of five (RO5), liquiritin complied with the criteria whereas glycyrrhizin showed violations. Lipinski parameters provide useful insight into the dermal penetration potential of small molecules, which in turn shape the nature and magnitude of their skin toxicity [24]. A compound that violates multiple Lipinski criteria, such as glycyrrhizin, may exhibit limited cutaneous permeation, yet may still produce localized irritation or inflammatory responses at the skin surface.

According to our network pharmacology, we identified three main targets, EGFR, STAT3, and SRC. In line with KEGG analysis, EGFR tyrosine kinase inhibitor resistance is functionally linked to mechanisms underlying skin sensitivity and toxicological responses. The EGFR tyrosine kinase signaling pathway is another critical regulator of epidermal homeostasis [31]. Aberrant EGFR activation or inhibition can disrupt keratinocyte proliferation, differentiation, and wound repair, leading to cutaneous adverse effects such as rash, dryness, and inflammation—a pattern frequently observed with EGFR-targeting agents [31]. From our KEGG analysis, the TRP (transient receptor potential) channels act as molecular sensors for thermal, chemical, and inflammatory stimuli in the skin [32]. Dysregulation of TRP channel activity, particularly TRPV1 and TRPA1, has been shown to induce neurogenic inflammation, erythema, and irritation, which are hallmark features of skin toxicity [32]. Increasing evidence indicates that dysregulation of STAT3 signaling in keratinocytes perturbs epidermal barrier homeostasis, thereby contributing to skin sensitivity and toxic outcomes under various stressors. Experimental models of atopic dermatitis and rosacea have shown that both overactivation and deficiency of STAT3 can aggravate barrier dysfunction and inflammation, underscoring STAT3-centered pathways as a critical mechanistic axis [33]. Notably, several enriched pathways—particularly TRP-channel regulation, EGFR signaling, and estrogen signaling—intersect with STAT3-mediated control of keratinocyte inflammation and barrier integrity, offering a mechanistic link to skin sensitivity and toxicity [33].

Our molecular docking supported these findings showing strong interactions of liquiritin and glycyrrhizin with proto-oncogene tyrosine-protein kinase (SRC). SRC is a non-receptor tyrosine kinase that plays a central role in various signaling pathways regulating cell proliferation, differentiation, survival, and inflammatory responses [34]. In the context of cutaneous biology, SRC activation has been implicated in keratinocyte hyperproliferation, epidermal barrier dysfunction, and inflammatory cytokine release, all of which are closely associated with skin sensitivity and toxicological reactions [35]. Several studies have demonstrated that SRC phosphorylation acts as a key mediator of skin inflammation and irritation, particularly through downstream activation of the MAPK/ERK and NF-κB pathways, leading to enhanced production of pro-inflammatory mediators such as IL-1β, TNF-α, and COX-2 [36]. In this study, both liquiritin and glycyrrhizin exhibited strong binding affinities with SRC in molecular docking analysis, suggesting that these compounds may modulate SRC-mediated signaling. Given SRC’s established involvement in epidermal inflammatory and stress responses, such interactions may underlie the observed differences in skin sensitivity and toxicity profiles between the two compounds.

Finally, the threshold of toxicological concern-based safety assessment suggests that liquiritin presents a favorable toxicological profile, with exposure levels remaining well below the established safety threshold for topical application. Although glycyrrhizin exposure marginally exceeded the TTC threshold, the predicted concentration is unlikely to elicit adverse effects under normal cosmetic use. These findings collectively indicate that both compounds exhibit acceptable safety margins, supporting their potential suitability for inclusion in dermal formulations. This finding is consistent with current practices, where licorice root extract is generally formulated at 0.1–1% in leave-on products [4,37].

It is important to acknowledge that the present in silico, database-driven toxicity assessment represents an early-stage predictive screening rather than a definitive toxicological evaluation. The primary objective of this analysis was to identify potential structural alerts and prioritize compounds for further investigation, rather than to establish conclusive evidence of toxicity. The novelty of this study lies in the establishment of an integrated HPLC–network pharmacology–TTC workflow, representing the first comprehensive framework that bridges analytical chemistry, computational toxicology, and quantitative risk assessment. By combining experimental compound identification through HPLC with in silico network pharmacology analysis and threshold of toxicological concern-based exposure evaluation, this approach enables a multidimensional assessment of both efficacy and safety within a single methodological pipeline.

GU contains a wide spectrum of flavonoids, chalcones, coumarins, and triterpenoids that extend beyond the two marker compounds evaluated in this study [15]. Although liquiritin and glycyrrhizin were selected due to their established toxicological relevance and substantial mechanistic evidence, minor constituents may also influence dermal responses or modulate the biological behavior of these markers. Reports on glabridin, isoliquiritigenin, and licochalcone derivatives illustrate that such compounds can exert antioxidant, prooxidant, or cell-modulating activities depending on context [15]. Therefore, a comprehensive safety evaluation of GU extract ultimately requires integration of untargeted HPLC profiling and in silico toxicity prediction for broader phytochemical classes. This study provides an initial framework focused on key toxicologically relevant markers, and future work should expand this approach to encompass the full chemical complexity of the extract. The absence of in vivo validation limits the direct extrapolation of these computational predictions to physiological contexts. Future studies incorporating in vitro assays and in vivo models will therefore be essential to confirm the predicted safety profiles and to elucidate the mechanistic pathways underlying the observed toxicity signals.

## 4. Materials and Methods

### 4.1. Preparation of Glycyrrhiza uralensis (GU) Root Extract and Reagents

Dried root of GU (3 Kg) was purchased from the National Development Institute of Korean Medicine. A total of 3.5 L of 70% ethanol (DAEJUNG, 023-2304, Gyeonggi, Republic of Korea) was added and extracted for 3 h at room temperature. The solvent was then filtered and separated using filter paper (Hyundai No. 2; cat. no. HD2-090). The filtrate was collected and concentrated under reduced pressure using a rotary vacuum evaporator, followed by freeze-drying to obtain the extract. Standard compounds, liquiritin and glycyrrhizin (purity 99%), were purchased from ChemFaces (Wuhan, China). Authentic standards were prepared in HPLC-grade water–methanol (1:1) at concentrations of 0.1 mg/mL and 1.0 mg/mL, respectively.

### 4.2. Identification and Quantification of Chemicals Contained in G. uralensis Root Extract

The freeze-dried extract was dissolved in methanol at a concentration of 10 mg/mL and analyzed by high-performance liquid chromatography (HPLC; Younglin 9100 series, Gyeonggi, Republic of Korea). Samples of *Glycyrrhiza uralensis* root extract, liquiritin, and glycyrrhizin were analyzed by HPLC with an injection volume of 20 μL each. The analytical conditions were as follows: a 30 min gradient elution from 5% to 100% aqueous acetonitrile containing 0.1% trifluoroacetic acid, using a Phenomenex Luna (Torrance, CA, USA) (2) C18 column (4.6 mm × 150 mm) at a flow rate of 1 mL/min. The detection wavelengths were set at 215. Both the column and sample compartment temperatures were maintained at 25 °C throughout the analysis.

To quantify liquiritin and glycyrrhizin in the *Glycyrrhiza uralensis* root extract, standard solutions of liquiritin (0.1 mg/mL) and glycyrrhizin (1.0 mg/mL) were first prepared and analyzed by HPLC under the same conditions as the sample extract. Retention times and peak areas for each standard were recorded to establish calibration data. The extract sample (10.0 mg/mL) was then injected, and the retention times were compared with those of the standards to confirm the identity of liquiritin and glycyrrhizin peaks. The concentrations of liquiritin and glycyrrhizin in the extract were calculated by comparing the peak areas of the extract sample with those obtained from the standard curves.

### 4.3. Analytical Method Validation

The linearity of the detector response was evaluated using calibration curves for liquiritin (6.25–1250 μg/mL) and glycyrrhizin (12.5–2500 μg/mL). The calibration equation was fitted as y=ax+b, where x is analyte concentration (μg/mL) and y is peak area. Ten-point calibration curves were constructed, with regression analysis performed to confirm linearity across the measured concentration range. Sensitivity was assessed following ICH guidelines, with a series of sample dilutions prepared in ethanol. The limit of detection (LOD) was defined as the lowest concentration producing a signal distinguishable from baseline noise (S/N = 3), and the limit of quantification (LOQ) as the lowest concentration where accurate and reliable measurement is assured (S/N = 10).

### 4.4. Structural Data Acquisition, Physicochemical Description, and In Silico Target Prediction

Chemical structures of identified compounds are obtained from PubChem (https://pubchem.ncbi.nlm.nih.gov/) in SMILES format. The physicochemical description was performed via SwissADME (http://www.swissadme.ch) in order to estimate oral bioavailability and permeability based on Lipinski’s rule of five. The analysis incorporated Lipinski’s “Rule of Five” to predict drug-likeness, focusing on key molecular properties including molecular weight (MW ≤ 500 Da), lipophilicity (logP ≤ 5), hydrogen bond donors (HBD ≤ 5), and hydrogen bond acceptors (HBA ≤ 10).

Chemical structures of identified compounds are obtained from PubChem (https://pubchem.ncbi.nlm.nih.gov/) in SMILES format. The targets associated with the compounds were predicted using SwissTargetPrediction (https://swisstargetprediction.ch/predict.php, accessed on 1 August 2025) and DrugBank database (https://go.drugbank.com/). Skin sensitivity and toxicology-related target genes in *Homo sapiens* were retrieved from the NCBI gene (https://www.ncbi.nlm.nih.gov/gene, accessed on 1 August 2025) and GeneCards (https://www.genecards.org/) databases to elucidate functional annotations and underlying biological mechanisms. Common intersecting targets between EC and influenza were identified using the Venny 2.1.0 web tool (https://bioinfogp.cnb.csic.es/tools/venny/, accessed on 1 August 2025) (Supplementary S1).

### 4.5. Network Construction and Analysis

Network pharmacology is a computational approach used to elucidate the pharmacological mechanisms of bioactive compounds by integrating compound–target and protein–protein interaction (PPI) networks. To investigate the interactions among the hub target proteins, a PPI network was generated using the Search Tool for the Retrieval of Interacting Genes/Proteins (STRING; https://string-db.org/, version 12.0) with a high confidence interaction score threshold of 0.700. The resulting interaction data were subsequently imported into Cytoscape (version 3.9.0) for topological analysis and visualization. Hub targets were identified based on network connectivity parameters, providing insight into potential key regulatory proteins.

### 4.6. Gene Ontology (GO) and Kyoto Encyclopedia of Genes and Genomes (KEGG) Pathway Enrichment Analysis

Gene Ontology (GO) and Kyoto Encyclopedia of Genes and Genomes (KEGG) pathway enrichment analyses were performed to elucidate the biological functions and signaling pathways associated with the identified targets. Functional annotation and enrichment analyses were conducted using the Database for Annotation, Visualization, and Integrated Discovery (DAVID; https://davidbioinformatics.nih.gov/). The enriched GO terms included biological processes (BP), molecular functions (MF), and cellular components (CC) revealing the physiological roles, biochemical activities, and subcellular localization of the identified targets. KEGG pathway analysis was performed to identify the biological pathways associated with the identified compounds, particularly those related to skin sensitivity and toxicological responses.

### 4.7. Prediction of Toxic Targets and Molecular Docking of Selected Compounds and Targets

The 3D crystal structures of targets were retrieved from RCSB protein data bank (https://www.rcsb.org/). Water molecules were removed, and polar hydrogen atoms were added to the targets using the Discovery Studio Visualizer. The 3D structure of selected compounds was downloaded from Pubchem database (https://pubchem.ncbi.nlm.nih.gov/) and further converted into PDB file using Avogadro software (ver 1.2.0). A ligand–target docking approach was used to evaluate binding affinity between ligand and target interaction for target specificity. Molecular docking was conducted using Pyrx (ver.0.9.6) via AutoDock Vina (ver.1.1.2) based on binding affinity (kcal/mol) scoring functions. The 2D ligand–target interaction was analyzed using the Discovery Studio Visualizer (v20.1).

### 4.8. Prediction of In Silico Skin Sensitivity and Irritation

The in silico assessment of skin sensitization and irritation potential for liquiritin and glycyrrhizin was conducted using the VEGA platform (https://www.vegahub.eu/, ver 1.2.4). The CONCERT model, a consensus-based quantitative structure–activity relationship (QSAR) system, was employed to predict the dermatotoxicological properties of the compounds. Each compound’s molecular structure was uploaded in SMILES format, and model reliability was evaluated according to the applicability domain defined within VEGA. The predictions were expressed as categorical outputs—“active” or “inactive”—for skin sensitization potential, and “irritating” or “non-irritating” for dermal irritation.

### 4.9. Cramer Class Assignment and Application for Threshold of Toxicological Concern (TTC) Thresholds

Essential SMILES codes for analysis were retrieved from PubChem and put into Toxtree package 3.1 to assign Cramer class I–III based on the extended decision tree to determine their toxicity potential [38]. The threshold of toxicological concern approach was used as a risk assessment tool when substance-specific toxicity data were not available. This threshold of toxicological concern value was applied as the reference point for risk characterization. The systemic exposure dose (SED) and the estimated daily intake (EDI) of compounds were calculated according to SCCS guidance for cosmetic ingredient safety assessment. The maximum allowable concentration (Cmax) of compounds in each product type was back-calculated to ensure that exposure remained below the threshold of toxicological concern for the Cramer class. For cosmetic use, we analyzed face cream scenarios in this study.

## Figures and Tables

**Table 1:** Linearity and sensitivity data of six 5-OH-PMF standards.

Analyte	Concentration Range (μg/mL)	Linear Regression Equation	*r* ^2^	LOD (μg/mL)	LOQ (μg/mL)
Liquiritin	6.25–1250	*y* = 573.21*x* − 1.3025	0.9994	1.5	6.25
Glycyrrhizin	12.5–2500	*y* = 388.75*x* + 0.5123	0.9997	3.1	12.5

**Table 2:** Lipinski’s molecular descriptors for liquiritin and glycyrrhizin.

Compound	MW (g/mol)	n-ROTB	HBA	HBD	MR	cLogP	TPSA
Liquiritin	418.4	4	16	5	101.67	0.4	145.91
Glycyrrhizin	822.93	7	9	8	202.84	1.49	267.04

MW—molecular weight (acceptable range: ≤500); n-ROTB: number of rotatable bonds (acceptable range: ≤10); HBA—hydrogen bond acceptor (acceptable range: ≤10); HBD—hydrogen bond doner (acceptable range: ≤5); MR—molar refractivity (acceptable range between 40–130); cLogP—lipophilicity (acceptable range: ≤5); TPSA—topological polar surface area (acceptable range: <140).

**Table 3:** Molecular docking analysis of liquiritin and glycyrrhizin.

Compound (Ligand)	Target (PDB ID)	Binding Affinity (kcal/mol)
Liquiritin	EGFR (4I23)	−9.7
	STAT3 (6TLC)	−8.1
	SRC (1YOJ)	−9.8
Glycyrrhizin	EGFR (4I23)	−9.0
	STAT3 (6TLC)	−9.5
	SRC (1YOJ)	−9.6

**Table 4:** Skin sensitization and irritation analysis using VEGA in silico platform.

Toxicity	Liquiritin	Glycyrrhizin
Skin sensitization	Inactive	Inactive
Skin irritation	Non-irritating	Non-irritating

**Table 5:** Risk assessment of liquiritin and glycyrrhizin as face cream.

Compound/ Product Type	A = Use Amount (g/day)	Cprod (%)	SED (µg/kg bw/day)	TTC (µg/kg bw/day)	Cmax (%)	Risk Characterization
Liquiritin /Face cream (leave-on)	1.54	0.011	1.41	1.5	0.0117	SED < TTC
Glycyrrhizin/ Face cream (leave-on)	1.54	0.015	1.93	1.5	0.0117	SED > TTC

## Data Availability

The original contributions presented in this study are included in the article. Further inquiries can be directed to the corresponding author.

## Supplementary Materials

The following supporting information can be downloaded at: https://www.mdpi.com/article/10.3390/ijms262311677/s1.
